# Dietary polyphenols maintain human health through modulation of gut microbiota

**DOI:** 10.3389/fphar.2025.1710088

**Published:** 2026-01-05

**Authors:** Mithun Rudrapal, André M. de Oliveira, Ravi Pratap Singh

**Affiliations:** 1 Department of Pharmaceutical Sciences, School of Biotechnology and Pharmaceutical Sciences, Vignan’s Foundation for Science, Technology and Research, Guntur, India; 2 Department of Environment Studies, Federal Centre of Technological Education of Minas Gerais (CEFET-MG), Contagem, Minas Gerais, Brazil; 3 Department of Pharmaceutical Sciences and Technology, Birla Institute of Technology, Ranchi, India

**Keywords:** dietary polyphenols, gut microbiota, polyphenol bioavailability, polyphenolmetabolites, health outcomes

## Abstract

The symbiotic interplay between dietary polyphenols and gut microbiota constitutes a focal point in contemporary scientific investigations, with profound impact in human health and diseases. The human gastrointestinal milieu serves as the locus for a diverse consortium of microbial organisms, collectively constituting the gut microbiota, which intricately modulate host metabolism, immune responses, and overall homeostasis. Dysregulation of gut microbial composition and functionality, known as dysbiosis, has been implicated in the progression of a plethora of gastrointestinal and systemic maladies, encompassing inflammatory bowel diseases, metabolic syndromes and neurological disorders, and so on. Polyphenols abundant in plant-derived food, exert multifaceted biological activities, encompassing antioxidative, anti-inflammatory and anticancer properties, among many others. These polyphenolic metabolites inextricably interact with the gut microbiota, exerting modulatory effects on microbial composition and functionality, thereby promoting the symbiotic physiological relationships between microbe and human host. In reciprocal fashion, the gut microbiota serves as pivotal vectors in the metabolism and bioavailability of polyphenols, engendering bioactive signalling metabolites which regulate systemic physiological effects and thereby maintain host health. This review emphasizes the imperative of comprehensively delineating an interplay between polyphenolic metabolites and gut microbiota in maintaining host health, while reflecting potential interventions of protective health outcomes in disease conditions.

## 1 Introduction

In recent years, the intricate symbiotic relationship between dietary polyphenols and human gut microbiota has emerged as a focal point in scientific investigations due to its profound implications in human health and diseases. The human gastrointestinal tract is host to a dynamic and densely populated microbial ecosystem, collectively referred to as the gut microbiota, which plays a pivotal role in modulating host physiology and biochemical metabolic systems (Jyoti and Dey, 2025). The human gut microbiota represents a highly heterogeneous consortium of microorganisms, encompassing bacteria, archaea, viruses, and fungi, which coexist in a dynamic equilibrium with the host (Nazir et al., 2024). This intricate microbial ecosystem exhibits a vast phylogenetic diversity, predominantly comprising members of the phyla *Firmicutes*, *Bacteroidetes*, *Actinobacteria*, and *Proteobacteria*, with distinct genera and species exerting differential functional roles within the gut environment. The composition and diversity of gut microbiota are subject to dynamic modulation by various endogenous and exogenous factors, including host genetics, dietary habits, environmental exposures, antibiotic usage, and lifestyle factors (Babaei, 2019; Townsend et al., 2021). Mounting evidence implicates dysbiosis, defined as dysregulation (alteration) in gut microbiota composition and functionality, in the pathogenesis of a myriad of gastrointestinal and extra-intestinal disorders (Singh et al., 2021). Moreover, emerging research demonstrates the role of dysbiosis in neurodegenerative disorders, autoimmune diseases, and microbial infections, highlighting the implications of gut microbiota dysregulation on host health and wellbeing (Alshehri et al., 2021; Nishida et al., 2021).

Polyphenols, a diverse group of secondary metabolites abundant in various plant-derived foods and beverages, have garnered significant attention for their multifaceted bioactivities, including antioxidant, anti-inflammatory, antimicrobial, and anticancer properties, among many others (Bolat et al., 2024). These bioactive metabolites exert their biological effects through some unambiguous mechanisms, including free radical scavenging action, modulation of cell signalling pathways, and enzyme activities (Joshi et al., 2020; Rudrapal et al., 2021). Of particular relevance to gut health, polyphenols possess the capacity to modulate gut microbiota composition and function through multiple mechanisms, including direct antimicrobial effects, prebiotic-like activities, and modulation of microbial enzymatic or signalling activities involved in polyphenol metabolism (Petersen and Mansell, 2025).

This comprehensive review aims to elucidate the complex interplay between gut microbiota and polyphenols, exploring their mechanistic interactions and implications in promoting or maintaining human health and protective functions against various pathological or disease conditions (Halliwell, 2024). The interaction between gut microbiota and dietary polyphenols holds significant implications for human health and diseases. Accumulated evidence suggests that polyphenol-rich diets or supplementation may exert beneficial effects on composition and function of gut microbiota, promoting the growth of commensal bacteria while inhibiting the proliferation of pathogenic species. Conversely, gut microbiota plays a pivotal role in the bioavailability and metabolism of polyphenols, contributing to the generation of bioactive metabolites with improved systemic effects on host physiology and metabolism (Cardona et al., 2013; Catalkaya et al., 2020; Mahdi et al., 2025). In the context of health and disease management, certain potential interventions that target gut microbiota include dietary modifications, dietary fibres, prebiotic formulations, dietary supplements which can help prevent and treat various gastrointestinal abnormalities and metabolic disorders (Ghanbari et al., 2024). Polyphenol-rich dietary interventions have been proposed as potential and adjunctive strategies to modulate gut microbiota composition and function, thereby ameliorating the risk of disease progression and promoting host health (Carding et al., 2015; Das and Nair, 2019; Hrncir, 2022; Schippa and Conte, 2014).

The objective of this review is to collate the interaction between dietary polyphenols and gut microbiota critically, as well as its impact on human health. This review emphasizes the mechanisms by which polyphenols are metabolized by gut microbiota, while exploring potential factors that limit the polyphenol’s effects, including bioavailability, metabolic factors, dietary content, and individual variability. Further, the review highlights how long-term consumption of polyphenols through diet and microbial adaptation affect the stability of gut microbiota and consequently health outcomes. This review integrates the current status of knowledge on polyphenol-gut microbiota interactions and in-depth scientific insights of mechanism of their biological effects in a variety of physiological or pathological disease conditions.

The search terms and keywords for the study selection were dietary polyphenols OR food polyphenols AND gut microbiota AND health outcomes AND bioavailability and metabolism AND polyphenol metabolites. The *in vitro* and *in vivo* (animal) studies along with pharmacological activity were used as further criteria for literature search. The search was carried out for last 15 years (2011–2025) of publications. Three independent reviewers (M.R., A.M.de O. and R.P.S.) conducted the literature search in the scientific databases and assessed/verified the eligibility of the studies based on the title and abstract. The inclusion criteria were (i) studies involving dietary or food polyphenols and their health outcomes, (ii) studies reported on bioavailability and metabolism of dietary polyphenols, (iii) studies performed in gut-microbiota-aided dietary polyphenol metabolites and their effects *in vitro* or *in vivo* models, (iv) interaction between dietary polyphenols and gut microbiotas, and (v) studies published from 2011 to 2025 (15 years, both years included). The exclusion criteria were (i) studies involving dietary polyphenols, chemistry, their antioxidant effects and role in disease management, (ii) studies on systematic reviews, meta-analysis and case reports, (iii) studies involving other than animal or human models, (iv) papers published before 2011, and (v) published articles in a language different from English. The published records cited in the manuscript were identified from the database search (PubMed Web of Science, Embase, ScienceDirect, Scopus) and other sources, considering inclusion and exclusion criteria.

## 2 Gut microbiota: functional roles, human health and disease management

The gut microbiota is a complex ecosystem consisting of trillions of microorganisms such as bacteria, viruses, fungi, and archaea that inhabit the gastrointestinal tract of humans and animals (Chakraborti, 2015). Recent research has shown that the gut microbiota plays a critical role in maintaining human health and preventing the development of various diseases (Jain et al., 2024; Neveu, 2010; Zhang S et al., 2024; Zhang et al., 2020) The gut microbiota is an essential component of the human body, and its role in maintaining human health has been the subject of extensive research in recent years. The gut microbiota can affect various physiological processes, including metabolism, immune function, and brain function, through several mechanisms such as fermentation of dietary substrates, production of short-chain fatty acids (SCFAs) (Kaur and Gupta, 2014), regulation of the gut barrier function, enhancement of nutrient absorption/metabolism, and modulation of the immune system (Cardona et al., 2013). The gut microbiota can also produce various vitamins, such as vitamin K and B vitamins, that are essential for human health. Moreover, the gut microbiota can influence the development of the immune system, particularly during early life, and protect against pathogenic microorganisms. Recent research has also shown that dysbiotic alterations in gut microbial communities lead to the development of various diseases, including inflammatory bowel disease (IBD), irritable bowel syndrome (IBS) (Zhang Y et al., 2024; Zuo and Ng, 2018), obesity (Ahmed et al., 2025; Anhê et al., 2015; Chakraborti, 2015), type 2 diabetes (Chang et al., 2025; Wang et al., 2020), colorectal cancer, and cardiovascular disease (Almeida et al., 2021; Mohsenzadeh et al., 2025; Novakovic et al., 2020; Zalila-Kolsi et al., 2025). The composition and dynamics of gut microbiota are subject to myriad influences, encompassing host genetics, environmental exposures, diet, lifestyle factors, and medical interventions (antibiotics usage) (Hasan and Yang, 2019). Therefore, maintaining a healthy gut microbiota is crucial for preventing the development of various diseases and promoting overall human health. Table 1 depicts functional roles of gut microbiota in maintaining human health and disease management.

**TABLE 1:** Prominent functional roles of microbiota in human health and diseases.

Microbiota	Role in human health	Role in diseases (dysbiosis)	References
*Bacteroides*	Aid in digestion, produce essential vitamins	Imbalance linked to obesity and diabetes	Li et al. (2015)
*Lactobacillus*	Maintain gut health, prevent infections	Reduced levels tied to gastrointestinal disturbances	Adeeb et al. (2018), Pärtty et al. (2018)
*Bifidobacterium*	Enhance immune response, inhibit pathogens	Low levels associated with irritable bowel syndrome (IBS)	Torres-Maravilla et al. (2022)
*Escherichia coli*	Produce vitamin K2, assist in digestion	Pathogenic strains cause foodborne illnesses	Singha and Gupta (2023)
*Firmicutes*	Ferment fibres, produce beneficial fatty acids	High proportions linked to obesity	Stojanov et al. (2020)
*Akkermansia muciniphila*	Support gut barrier function	Decreased levels linked to metabolic disorders	Bahena-Román et al. (2022)
*Faecalibacterium prausnitzii*	Produces anti-inflammatory metabolites	Low levels associated with inflammatory bowel disease (IBD)	Hansen et al. (2012)
*Prevotella*	Ferment dietary fibers to produce short-chain fatty acids (SCFAs)	High levels linked to inflammation and rheumatoid arthritis	Jiang et al. (2022)
*Clostridium difficile*	Part of normal gut flora in small numbers	Overgrowth can lead to severe colitis and diarrhoea	Babu et al. (2024)
*Roseburia*	Produces SCFAs	Reduced levels associated with IBS and IBD	Kleiman et al. (2015)
*Enterococcus faecalis*	Helps with nutrient absorption	Overgrowth linked to urinary tract infections and endocarditis	Calderón-Parra et al. (2022)
*Methanobrevibacter smithii*	Involved in methane production in the gut	High levels associated with constipation	Ghoshal et al. (2016)
*Ruminococcus*	Degrade complex carbohydrates	Imbalance linked to gut disorders like IBS	Zhai et al. (2023)
*Bacteroides fragilis*	Modulates immune system, protects against pathogens	Overgrowth associated with colorectal cancer	Scott et al. (2022a)
*Veillonella*	Metabolizes lactate into SCFAs	Imbalance linked to exercise-induced gut problems	Kostic, (2018)
*Streptococcus thermophilus*	Aids in lactose digestion, used in probiotics	Imbalance can lead to respiratory and skin infections	Renye et al. (2023)

The gut microbiota has been demonstrated to exert a significant influence on cognitive functions through the complex network known as the gut brain axis (Matiş et al., 2023a). The administration of specific probiotics referred to as psychobiotics has shown improvement in key neuropsychiatric manifestations such as hyperactivity aggression and concentration in paediatric populations with gastrointestinal disorders. This cognitive modulation is largely mediated by the microbiotas ability to regulate fundamental neurotransmitters for instance certain gut bacteria are involved in the synthesis of serotonin as a key regulator of mood and satiety and gamma-aminobutyric acid (GABA) as a primary inhibitory neurotransmitter crucial for calming neural activity and managing stress. Furthermore, the microbiota helps modulate the hypothalamic pituitary adrenal (HPA) axis thereby influencing cortisol levels and the body´s stress response which is intrinsically linked to cognitive performance and emotional regulation. Beyond neurotransmitter balance, gut microbes can produce neuroactive metabolites like short chain fatty acids which possess anti-inflammatory properties and can influence neurogenesis and blood brain barrier (BBB) integrity. Thus, the gut microbiota acts as a pivotal endocrine organ that communicates with the brain via neural immune and hormonal pathways ultimately shaping cognitive processes behaviour and overall mental health. The microbiome also plays a significant role in alleviating neuropsychiatric disorders. In experimental animals, probiotic treatment subdues anxiety, such as supplements included strains of *Lactobacillus*, *Bifidobacterium*, and *Streptococcus* reduced significantly neuropsychiatric illness (Matiş et al., 2023b).

## 3 Dietary polyphenols: sources and biological significance

Plant-based diet have health promoting effects that are largely attributed to their bioactive phytochemicals (Samtiya et al., 2021; Szabo et al., 2021). Polyphenols abundant in plant-based food are a diverse group of phytochemicals, which include flavonoids, phenolic acids, lignans, stilbenes, catechins, and anthocyanins (Rudrapal and Chetia, 2016). Dietary polyphenols in particular have been implicated to play significant role in preventing various chronic diseases, including cardiovascular diseases, neurodegenerative disorders, diabetes, inflammatory illness, and certain types of cancer, and infectious diseases (Gasmi et al., 2022; Samtiya et al., 2021). Polyphenols are found in a wide range of food plants, including vegetables, fruits, pulses, cereals, grains, nuts, herbs, spices, and teas (Rudrapal et al., 2022a).

Dietary polyphenols are a diverse group of metabolites that are widely distributed in plant-based foods (Manach et al., 2004; Rudrapal et al., 2024; Rudrapal et al., 2022b) and have been shown to have various biological activities, including antioxidant, anti-inflammatory, cardioprotective, neuroprotective, and anticancer properties (Rudrapal et al., 2021). They play a significant role in promoting and maintaining human health. Table 2 presents the sources and biological significance of some representative dietary polyphenols.

**TABLE 2:** Sources and biological roles of dietary polyphenols.

Dietary polyphenol	Food source	Biological roles
Vanillin	*Vanilla planifolia* (vanilla orchid) (Yang et al., 2017)	Antioxidant (Tai et al., 2011), Antidiabetic (Tai et al., 2011), Neuroprotective (Salau and Shahidul Islam, 2024)
Ferulic acid	Tomatoes, sweet corn, rice bran (Kapcum et al., 2016), whole-grain, citrus fruits, banana, coffee, orange, eggplant, bamboo shoots, beetroot, cabbage, spinach, and broccoli (Zhao and Moghadasian, 2008)	Antihypertensive, Anti-insulin resistance (Naowaboot et al., 2018), Anticancer (Gao et al., 2018), Neuroprotective (Dong and Huang, 2022), Antidiabetic (Li et al., 2022), Cardioprotective (Pandi et al., 2022)
Caffeic acid	Propolis, olives, coffee beans, fruits, and vegetables (Verma and Hansch, 2004)	Antidiabetic (Salau et al., 2022), Anticancer (Rajendra Prasad et al., 2010), Neurodegenerative (Zaitone et al., 2018)
(−)-Epicatechin	Green tea (Saito et al., 2006), grape (Iacopini et al., 2008), cocoa (Iacopini et al., 2008)	Antidiabetic (Abdulkhaleq et al., 2017; Paramanik et al., 2025), anticancer ()
Epigallocatechin-3-gallate (EGCG)	Green tea (Azam et al., 2004)	Anticancer (Azam et al., 2004), antiobesity (Moon et al., 2007), Neuroprotective (Zhang S et al., 2020), Anti-infective (Steinmann et al., 2013)
Quercetin	Grapes (Iacopini et al., 2008), onion, asparagus, berries, apple, broccoli, Chili pepper, kale, leek, lettuce, spinach, chive (Dabeek et al., 2019)	Cardioprotective (Liu et al., 2021), Anticancer (Wu et al., 2019), Antidiabetic (Vessal et al., 2003), Neuroprotective (Cho et al., 2006), Antihypertensive (Edwards et al., 2007), Antimicrobial (Hirai et al., 2010)
Rutin	Grape (Iacopini et al., 2008), tea, green asparagus, onions, buckwheat, wine, eucalyptus, apples and berries (Riva et al., 2020)	Antidiabetic (Fernandes et al., 2010), Neuroprotective (Nassiri-Asl et al., 2008), Antiobesity (Yuan et al., 2017), Cardioprotective (Li et al., 2013), Anticancer (Chang et al., 2020), Antihypertensive (Oyagbemi et al., 2020)
Resveratrol	Grapes (Iacopini et al., 2008)	Neuroprotective (Hou et al., 2018), Anticancer (Opipari et al., 2004), Cardioprotective (Opipari et al., 2004), Antidiabetic (Do et al., 2012), Anti-infective (Cao et al., 2011)
Hesperetin	Citrus fruits (M. Singh et al., 2008)	Anticancer (Alshatwi et al., 2013), Anti-infective (Iranshahi et al., 2015), Antidiabetic (Chen et al., 2019), Neuroprotective (Muhammad et al., 2019), Cardioprotective (He et al., 2017)
Daidzein	Soybean (Jang et al., 2020)	Anticancer (Sathyamoorthy and Wang, 1997), Antidiabetic (Laddha and Kulkarni, 2021), Cardioprotective (Ahmad et al., 2024), Neuroprotective (Aras et al., 2015)
Naringenin	Citrus fruits, including grapes, oranges, blood oranges, lemons, and grapefruit (Cai et al., 2023; Hartogh and Tsiani, 2019)	Antioxidant and anti-inflammatory (Cai et al., 2023), cardioprotective (Testai et al., 2017), neuroprotective (Nouri and Chen, 2019), anticancer (Choi et al., 2020)
Genistein	Soy-based foods, broad beans and chick peas (Spagnuolo et al., 2015)	Anticancer (Tuli et al., 2019), menopausal symptoms (Thangavel et al., 2019), anti-bone and cartilage diseases (Wu and Liu, 2022)
Myricetin	Fruits, vegetables, berries, teas, wine (Agraharam et al., 2022; Imran et al., 2021)	Antioxidative properties and prooxidative properties (Ong and Khoo, 1997), antidiabetic, anticancer, immunomodulatory, cardiovascular, analgesic and antihypertensive (Taheri et al., 2020)

## 4 Dietary polyphenols and gut microbiota: interaction and impact on health

Polyphenols influence the dynamics of resident gut microbiota and a positive relationship between the two can promote human health and the prevention of diseases. Recent research suggest that dietary polyphenols can influence the gut microbiota composition and function, which may contribute to their health benefits (Rodriguez-Mateos et al., 2024; Selma and Tomás-Barberán, 2009; Tomás-Barberán et al., 2016; Wang et al., 2022). Gut microbiota facilitate the cardioprotective (Anselmi et al., 2021), neuroprotective effects (Pan et al., 2021; Rudrapal et al., 2024), antidiabetic/antiobesity (Gowd et al., 2019), anticancer (Westfall et al., 2021), and antimicrobia (Rudrapal et al., 2022c) activities of these polyphenols by producing polyphenol metabolites which protect gut barrier integrity, mitigate cellular oxidative stress and serve protective functions against inflammations/immune reactions and associated cellular damages.

Polyphenols are high molecular weight aromatic metabolites comprising complicated chemical (bulky) structures which impedes their absorption in the intestine. Gut microbiota, however, breakdown these polyphenols into low molecular weight metabolites which can be readily absorbed in the intestine (Wang et al., 2022). Interaction of polyphenols with the gut microbiota thus play a significant role in the health benefits of these polyphenols as it aids their breakdown to useful and easily absorbable metabolites. The impact of dietary polyphenols on gut microbiota can vary depending on several factors such as the type and amount of polyphenol intake through food, the host’s genetic makeup, and the gut microbiota composition. However, during microbiome dysbiosis, interaction between polyphenols and microbiome may have substantial implications for prevention or protective functions against diseases including cardiovascular diseases (CVDs), diabetes, obesity cancer, neurodegenerative disorders, infectious illness, and immune-inflammatory diseases (Vamanu and Rai, 2021).

### 4.1 Interaction between dietary polyphenols and gut microbiota

**FIGURE 1:**
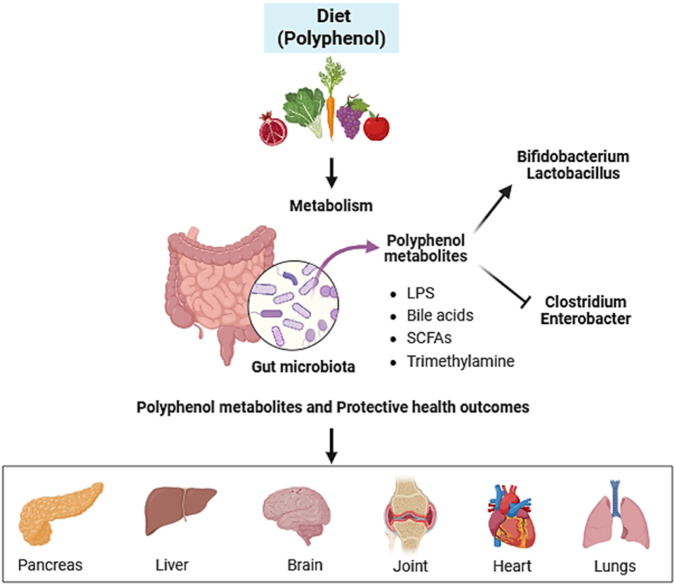
Interaction between dietary polyphenols and gut microbiota and resulting polyphenol metabolites and their impact on human health. This diagram illustrates the bidirectional relationship between the consumption of dietary polyphenols and the gut microbiota ecosystem. Dietary polyphenols (top panel) are metabolized by the gut microbiota, a process that modulates the composition of microbial communities. Specifically, there is a promotion of beneficial bacteria (such as Bifidobacterium and *Lactobacillus*) and an inhibition of potential pathobionts (such as *Clostridium* and *Enterobacter*). As a result of this interaction, various bioactive metabolites are generated (central panel), including short-chain fatty acids (SCFAs), bile acids, lipopolysaccharides (LPS), and trimethylamine. These metabolites, along with the polyphenols themselves and their microbial transformation products, exert systemic effects. They positively influence the function of key organs and systems (bottom panel), such as the liver, pancreas, brain, joints, lungs, and heart, contributing to protective health outcomes. Created with BioRender.

The interaction between dietary polyphenols and gut microbiota is a complex process that involves several mechanisms such as absorption, biotransformation, and metabolism. Polyphenols can interact with gut bacteria by binding to their cell membranes or extracellular matrix, which can influence bacteria’s growth and metabolism (Lacombe et al., 2013; Tao and Chen, 2019). Polyphenols can also undergo biotransformation in the gut by the gut microbiota, which can result in the production of metabolites that may have health promoting effects. Figure 1 depicts the interaction between dietary polyphenols and gut microbiota and resulting polyphenol metabolites and their impact on human health.

### 4.2 Bioavailability and metabolism of dietary polyphenols by gut microbiota

The bioavailability of dietary polyphenols can vary significantly due to factors such as the type and the number of polyphenols ingested through diet, the host’s genetic makeup, and the composition of the gut microbiota (Lippolis et al., 2023). Various types of polyphenols, including flavonoids, phenolic acids, and lignans, have diverse chemical structures with complex physicochemical properties such as water solubility, lipophilicity, etc., That affect their absorption and metabolism. Moreover, higher concentrations of ingested polyphenols may saturate the absorption mechanisms, leading to reduced bioavailability. Individual genetic differences also influence the enzymes involved in polyphenol metabolism, thereby affecting their bioavailability and biological effects (Del Rio and Crozier, 2013; Pasinetti et al., 2018). The diversity and composition of the gut microbiota are also key determinants in polyphenol’s bioavailability.

The gut microbiota plays a crucial role in the bioavailability of dietary polyphenols by converting complex polyphenols into simpler metabolites that are more bioavailable and possess different biological activities. Due to the poor absorption in the upper gut, polyphenols enter into the colon, where they undergo microbial fermentation into bioactive metabolites (Bié et al., 2023). For instance, epigallocatechin gallate (EGCG), a prominent polyphenol in green tea, has low bioavailability due to poor absorption in the small intestine. Recent studies have shown that gut microbiota can convert EGCG into simpler phenolic acids such as 5-(3′,4′-dihydroxyphenyl)-gamma-valerolactone (M5) and 5-(3′-Hydroxyphenyl)-gamma-valerolactone (M4), which exhibit significant anti-inflammatory and anticancer properties (Marín and Lombó, 2015; Zhang S et al., 2024). Isoflavones, including genistein and daidzein present in soy products, beans and certain vegetables, are partially absorbed from the small intestine, with the daidzein being converted in the colon by gut bacteria to equol, a metabolite with potent estrogenic and anti-inflammatory properties (Wu and Liu, 2022). Some lignans, such as secoisolariciresinol diglucoside present in flaxseeds, sunflower seeds, sesame seeds, pumpkin seeds undergo conversion in the gut to enterolignans that include enterolactone and enterodiol, both of which exhibit high-spectrum health-promotional effects as antioxidant, anti-inflammatory, neuroprotective, hypolipidemic and antimicrobial agents (Senizza and Lucini, 2020). Following the intake of polyphenols in red wine (obtained from red grapes), gut microbiota convert flavan-3-ols into the bioactive metabolite 3-hydroxyphenylpropionic acid, thus contributing to cardiovascular health (Cueva et al., 2017). Similarly, ellagitannins (berries and pomegranates) are poorly absorbed in the small intestine and reach the colon relatively intact, where they are metabolized by gut bacteria into bioactive products called urolithins, which exert potent anti-inflammatory and anticancer activities (Banc et al., 2023). Rastmanesh reported (Rastmanesh, 2011) that polyphenols with high bioavailability and promotion of the activity of certain gut microbiomes aid weight loss in obese individuals.

The metabolism of polyphenols by gut microbiota has profound implications for health and disease management. Research have identified several gut microbial species involved in polyphenol metabolism. *Akkermansia muciniphila*, for instance, metabolizes ellagic acid into urolithins, which possess anti-inflammatory and anticancer effects. *Faecalibacterium prausnitzii* is known for metabolizing flavonoids into anti-inflammatory metabolites that support gut health. Similarly, *Bacteroides fragilis* can convert complex polyphenols such as quercetin into simpler metabolites, bioavailable metabolites that enhance immune modulation (Mithul Aravind et al., 2021). Cardiovascular health, for example, benefits from metabolites like 3,4-dihydroxyphenylacetic acid derived from catechins (tea polyphenol), which improve vascular function and reduce oxidative stress. In cancer prevention, metabolites such as urolithins from ellagic acid and valerolactones from EGCG modulate cell proliferation and induce apoptosis, exhibiting strong anticancer properties (Pourová et al., 2018). Additionally, phenolic acids produced from quercetin and resveratrol offer potent anti-inflammatory effects, which are crucial in managing chronic inflammatory diseases (Catalkaya G. & Capanoglu, 2020). Resveratrol goes through metabolic processing in the colon towards dihydroresveratrol, which increases benign bacteria like Bacteroidetes and *Lactobacillus*, decreases pathogenic bacteria like *Enterococcus faecalis*, and eventually reduces inflammation via NF-κB signaling (Chaplin and Mercader, 2018). Similarly, anthocyanins (berries, grapes, radishes, red potatoes, cabbage) are poorly absorbed and mostly converted into phenolic acid, such as protocatechuic acid, due to gut microbiota, contributing to the growth of beneficial bacteria like *Faecalibacterium* and *Lactobacillus* (Kasprzak-Drozd et al., 2021). EGCG (green tea) is partly absorbed into the small intestine, whereas the rest gets metabolized by gut microbiota (*Lactobacillus*, *Bacteroides*) into various kinds of phenolic acids which in turn enhances *bifidobacteria* and inhibits pathogens like *Clostridium difficile* (Pérez-Burillo and Rufián-Henares, 2021).

The biotransformation of polyphenols can involve various microbial enzymes and pathways, such as esterases, glucuronidases, and ring-fission pathways (Makarewicz et al., 2021). A study with a symbiotic (a combination of probiotics and polyphenol-rich prebiotics) composed of a grape-derived prebiotic known as the Bioactive Dietary Polyphenol Preparation (BDPP) and a combination of the probiotics *Lactobacillus plantarum* and *Bifidobacterium longum*, is described to attenuate the chronic-stress induced inflammatory responses in the ileum and the prefrontal cortex (Westfall et al., 2021). Pharmacokinetic studies substantiate that the effect may be attributed to specific synbiotic-produced metabolites including gallic acid (GA), 4-hydroxycinnamic acid (4-HCA), homovanillic acid (HVA), 3-hydroxyphenylpropionic acid (3-HPPA), 4-HPPA, 3-hydroxyphenylacetic acid (3-HPAA), the resveratrol microbial metabolite dihydroresveratrol (DHRSV), and the characteristic flavonoid microbial metabolite 5-(3′,4′-dihydroxyphenyl)-γ-valerolactone (DHVL).

**FIGURE 2:**
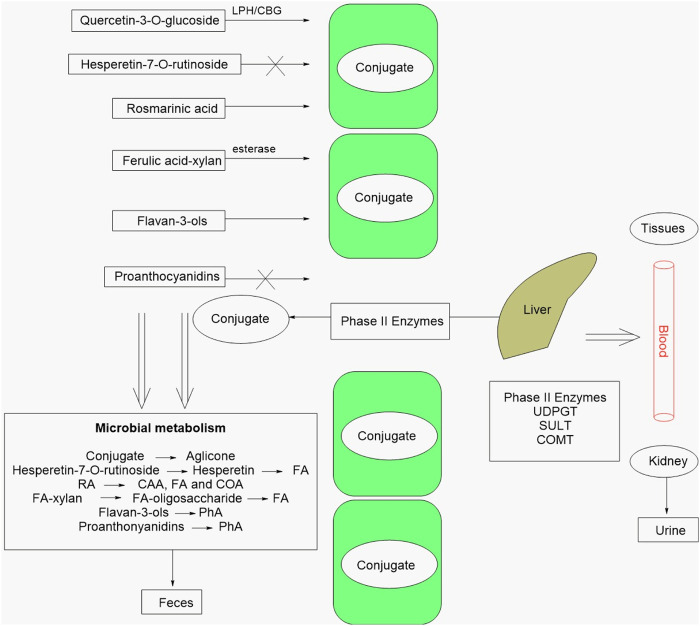
Metabolic pathways of selected polyphenols in the organism. This scheme details the primary biotransformation pathways of polyphenols, encompassing both human metabolism (conjugation phase) and intestinal microbial metabolism (breakdown phase). In the small intestine, human enzymes such as lactase phlorizin hydrolase (LPH) and cytosolic β-glucosidase hydrolyze complex polyphenols (conjugates, such as glycosides) into their simpler forms (aglycones). Subsequently, conjugation enzymes like UDP-glucuronosyltransferase (UDPGT), sulfotransferases (SULT), and catechol-O-methyltransferases (COMT) in the liver and other tissues chemically modify these compounds to facilitate their excretion. In the large intestine, the gut microbiota plays a crucial role in metabolizing these compounds. The diagram exemplifies the transformation of specific polyphenols into bioactive phenolic acids: 1. Hesperetin-7-O-rutinoside (found in citrus) is converted to hesperetin and finally to ferulic acid (FA). 2. Rosmarinic acid (RA) is metabolized into caffeic acid (CAA) and then into FA. 3. Proanthocyanidins (condensed tannins) and flavan-3-ols are degraded to phenylacetic acid (PhA). These final phenolic acids are key metabolites, widely recognized for their beneficial health effects. Adapted, by permission (Creative Commons Attribution 3.0 License), from (Vladimir-Knežević et al., 2012). LPH, cytosobetaglucosidase, LPH, lactase phlorizin hydrolase, UDPGT, UDP glucuronosyl transferase, SULT, Phenol sulfotransferase, COMT, Cathechol-O-methyltransferase, FA, ferulic acid, RA, rosmarinic acid, CAA, caffeic acid, COA, m-coumaric acid, PhA, Phenylacetic acid.

Polyphenols are metabolized in the gut through several biotransformation pathways, including hydrolysis, dehydroxylation, demethylation, and reduction, resulting in the production of metabolites with different biological activities (Stevens and Maier, 2016) (Figure 2).

The metabolism of polyphenols by the gut microbiota can also affect the composition and function of the gut microbiota itself. Gut microbiota plays a crucial role in the biotransformation of polyphenols as they can convert complex polyphenols into simple metabolites that are more bioavailable and have different biological activities. For instance, flavan-3-ols, which are abundant in tea, grapes, and cocoa (Van Dorsten et al., 2012; Zhou et al., 2019), can be converted by the gut microbiota into phenolic acids and urolithins, which have been shown to have anti-inflammatory and anticancer properties (Tzounis et al., 2008). Similarly, quercetin, a flavonoid found in onions, apples, and berries, can be converted by the gut microbiota into isoflavones, which have been shown to have estrogen-like properties and may play a role in preventing osteoporosis and cardiovascular diseases (Al-Ishaq et al., 2021). Polyphenols are present in plants in various forms, like aglycones (absorbed from the small intestine) and glycosides, esters or polymers (hydrolysed by intestinal enzymes) or by the colonic microflora. Glycosides can be hydrolysed through two possible ways: the first one involves the action of lactase phlorizin hydrolase (LPH) in the brush-border of the small intestine epithelial cells, so that the aglycone forms may then enter epithelial cells by passive diffusion due to increased lipophilicity. The second way involves cytosolic betaglucosidase (CBG) within the epithelial cells, that lead the polar glucosides to be transported through the active sodium-dependent glucose transporter 1 (SGLT1). SLGT1 is known not to transport flavonoids but the glycosylated flavonoids, and some aglycones, can inhibit the glucose transporter (Vladimir-Knežević et al., 2012).

**FIGURE 3:**
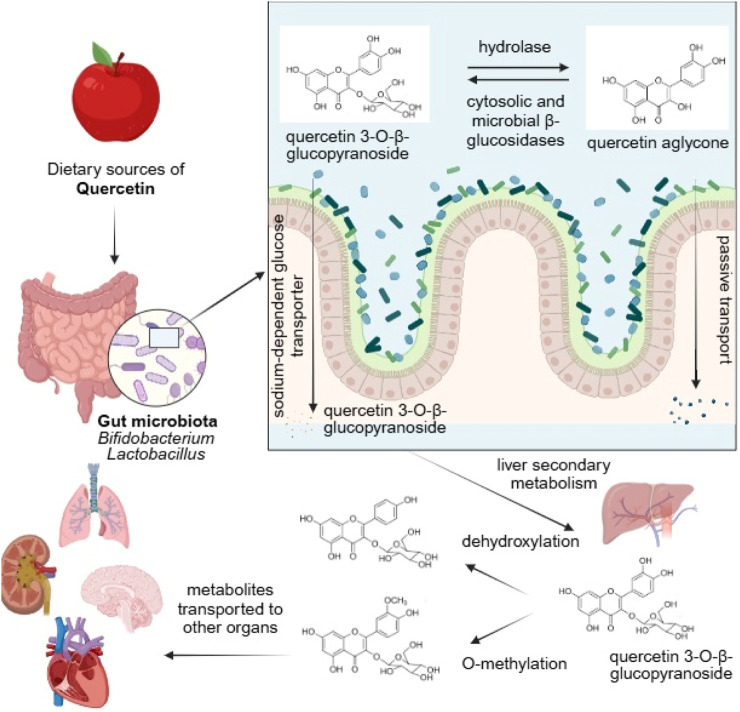
Biotransformation and metabolism of quercetin by gut microbiota. This figure illustrates the multi-step process by which the dietary flavonoid quercetin is processed within the human body, highlighting the essential role of the gut microbiota. The process begins with dietary sources of quercetin, often consumed in a glycosylated form (e.g., quercetin 3-O-β-glucopyranoside). In the small intestine, this compound can follow two initial pathways: 1) It can be directly absorbed via the sodium-dependent glucose transporter (SGLT1), or 2) it can be hydrolyzed by bacterial or human hydrolases and β-glucosidases to release the active quercetin aglycone. Subsequently, the aglycone can be absorbed and undergo secondary metabolism in the liver, where it is modified through processes like O-methylation and dehydroxylation to form various metabolites. Simultaneously, the gut microbiota (including genera like *Bifidobacterium* and *Lactobacillus*) plays a crucial role in further breaking down the quercetin aglycone and its metabolites through additional dehydroxylation and ring-fission reactions, producing simpler phenolic acids and other small molecules. These final quercetin-derived metabolites are then transported to various organs throughout the body, where they exert their localized biological effects and contribute to the observed health benefits associated with quercetin intake. Created with Biorender.

The metabolism of polyphenols in the gut may be a complex process, involving the metabolic degradation of anthocyanins being better known than the others. The bacterial metabolism involves the cleavage of glycosidic linkages and breakdown of anthocyanidin heterocycle (from C-ring), and degradation into phloroglucinol derivatives (from A-ring) and benzoic acids (from B-ring) (Aura et al., 2005; Fernandes et al., 2015). O-demethylation is also described (Keppler, 2005). Figure 3 depicts biotransformation and metabolism of quercetin by gut microbiota. The figure shows how the process begins with two initial pathways: absorbtion via the sodium-dependent glucose transporter (SGLT1), or hydrolyzation by bacterial or human hydrolases and β-glucosidases to release the active quercetin aglycone.

Once absorbed by the gastrointestinal tract, quercetin is converted into a carbohydrate conjugate by gut microbiome bacteria of the genus *Bifidobacterium* and *Lactobacillus*. The conjugated form crosses the membrane with the help of a sodium-dependent glucose transporter, and subsequently undergoes secondary metabolism in the liver. The metabolites are then transported to the major organs of the human body (heart, kidneys, lungs, central nervous system), where they exert their effects.

Polyphenols with polymeric nature and high molecular weight like proanthocyanidins are poorly absorbed, which causes high concentrations in gastrointestinal tract lumen and have direct effects on the intestinal mucosa. This effect is especially important when the intestine is exposed to oxidizing agents or is affected by inflammation and diseases such as cancer. These metabolites are, therefore, carried to the colon where they undergo structural modifications, those absorbed in the upper part of the gastrointestinal tract being metabolized in the liver and excreted in the bile or directly from the enterocyte back to the small intestine and reaching the colon but in a different form (e.g., glucuronide conjugates). The colonic microflora hydrolyses glycosides into aglycones and extensively metabolizes the aglycones into simpler metabolites, such as phenolic acids. However, conjugation and metabolism help to attenuate the potential toxic effects that accomplish polyphenols absorption. The main conjugation mechanisms include glucuronidation, sulfation andmethylationcatalyzed by UDP glucuronosyl transferase (UDPGT), phenol sulfotransferase (SULT) and catechol-O-methyl transferase (COMT) (Scalbert and Williamson, 2000).

### 4.3 Interrelationship among dietary polyphenols, gut microbiota and health implications

The consumption of dietary polyphenols has been associated with several health benefits, such as the prevention of chronic diseases including cancer (Karam et al., 2025; Y. Li et al., 2025; Tian et al., 2016; You et al., 2025), cardiovascular diseases (Alotaibi et al., 2021; Bhagani et al., 2020; Chen X et al., 2025; Chen Y. Y. et al., 2025; Liu Q et al., 2025; Mansour et al., 2024), metabolic disorders, and neurodegenerative diseases (Di Meo et al., 2020; Ross et al., 2024; Shi et al., 2024). These health benefits may be mediated by the interaction between dietary polyphenols and gut microbiota. Polyphenols can also modulate the gut microbiota metabolism by promoting the production of metabolites with different biological activities (Rodríguez-Daza et al., 2021). Polyphenols can modulate the gut microbiota composition by promoting the growth of beneficial bacteria such as *Bifidobacterium* and *Lactobacillus* and inhibiting the growth of pathogenic bacteria such as *Clostridium* and *Enterobacter*. Tea polyphenols including epicatechin inhibit the growth of *Clostridium* and *Enterococci*. Polyphenols can also modulate the gut microbiota function by altering the expression of microbial genes involved in various metabolic pathways, such as carbohydrate metabolism and amino acid metabolism (Anhê and Marette, 2017; Martín-Peláez and Fitó, 2017). For example, resveratrol, a polyphenol found in grapes and red wine, has been shown to promote the growth of *Lactobacilli* and *Bifidobacteria* (Qiao and Le, 2014) and inhibit the growth of pathogenic bacteria such as *Escherichia coli* (Vestergaard and Ingmer, 2019). Resveratrol can also promote the production of SCFAs such as butyrate (Tain et al., 2021), which have been shown to have anti-inflammatory and anticancer properties. Likewise, dietary quercetin has been known to alter gut microbial composition because of its probiotic-like activity and promotion of certain bacteria. Similarly, chlorogenic acid assists in improving resistance against intestinal inflammation, oxidative stress, and gut microflora disorder caused by infections of *Clostridium perfringens type A* and *Salmonella pullorum* (Rana et al., 2022; Zeng et al., 2022). Ellagitannins, for example, abundant in pomegranates and strawberries, can be converted by the gut microbiota into urolithins, which have been shown to have anti-inflammatory and anticancer properties (Bialonska et al., 2009). Urolithins can also promote the growth of beneficial bacteria such as *Akkermansia muciniphila*, which can protect against obesity and diabetes. In a recent study, a black tea and or a red wine grape extract (RWGE), both containing complex mixtures of dietary polyphenols have been investigated with the help of simulator of the intestinal microbial ecosystem (SHIME), an *in vitro* ecosystem of gut microbiota. Black tea stimulated *Klebsiella* and enterococci and reduced *B. coccoides* (bifidobacteria) and *Victivallis.* On the other hand, RWGE promoted the growth of *Klebsiella*, *Cloacibacillus*, and *Victivallis*, while it decreased *B. coccoides* (bifidobacteria), *Anaeroglobus*, and *Bacteroides* (Kemperman et al., 2013). Table 3 illustrates specific polyphenols, their metabolites and impact on human health.

**TABLE 3:** Specific polyphenol metabolites and their impact on human health.

Polyphenol	Food sources	Metabolites produced by gut microbiota	Associated microbiota	Impact on human health	References
Quercetin	Apples, Onions, Green tea	Phenylacetic acid, 4-hydroxyphenylacetic acid	*Bacteroides*, *Clostridium*, *Eubacterium*	Anti-inflammatory antioxidant, supports cardiovascular health	Bondonno et al. (2015), Shorobi and Rahman (2023)
Epicatechin	Green tea, Apples, dark chocolate	3,4-Dihydroxyphenylacetic acid, 3-hydroxyphenylacetic acid	*Bifidobacterium*, *Lactobacillus*, *Eubacterium*	Improves vascular function, cardioprotective, Antioxidant	Dower and Hollman (2015)
Resveratrol	Red grapes, Peanuts, Berries	Dihydroresveratrol, lunularin	*Bacteroides*, *Clostridium*, *Lactobacillus*	Anti-inflammatory, cardioprotective, anticancer	Chen et al. (2022), Wu and Hsieh (2011)
Catechin	Green tea, Apples, Dark chocolate	3-(3,4-Dihydroxyphenyl)-propionic acid, 3-Hydroxybenzoic acid	*Lactobacillus*, *Bifidobacterium*, *Eubacterium*	Enhances metabolic health, reduces blood pressure	Elbarbry et al. (2022)
Anthocyanins	Berries, Red cabbage, Black rice	Protocatechuic acid, hloroglucinol aldehyde	*Bifidobacterium*, *Lactobacillus*, *Enterococcus*	Antioxidant, supports eye health, anti-inflammatory	Oliveira et al. (2020), Yudina and Khlestkina, (2021)
Curcumin	Turmeric, Ginger	Dihydroferulic acid, Ferulic acid	*Bacteroides*, *Clostridium*, *Eubacterium*	Anti-inflammatory, supports brain health, anticancer	Tsuda (2018)
Ellagic acid	Pomegranate, Berries, Nuts	Urolithin A, Urolithin B	*Bacteroides*, *Clostridium*, *Roseburia*	Antioxidant, anti-inflammatory, supports gut health	Gupta et al. (2021), Mannino et al. (2023)
Ferulic acid	Whole Grains, Rice, Apples	Vanillic acid, Hydroferulic acid	*Bacteroides*, *Clostridium*, *Lactobacillus*	Antioxidant, supports skin health, anti-inflammatory	Drăgan et al. (2018), Sun and Shahrajabian (2023)
Chlorogenic Acid	Coffee, Apples, Blueberries	Hippuric acid, caffeic acid	*Bacteroides*, *Clostridium*, *Eubacterium*	Antioxidant, supports cardiovascular health, antidiabetic	Yuan et al. (2017)
Rutin	Buckwheat, Citrus fruits, Apples	Quercetin, 3,4-Dihydroxyphenyl acetic acid	*Bifidobacterium*, *Lactobacillus*, *Eubacterium*	Antioxidant, supports vascular health, anti-inflammatory	Chen et al. (2022), Diwan and Gobe (2017)
Genistein	Soybeans, Soy products	*p*-Ethylphenol, Equol	*Bacteroides*, *Clostridium*, *Lactobacillus*	Supports bone health, anticancer	Gupta and Sharma (2023), Skrajnowska et al. (2024)
Naringenin	Citrus fruits, Tomatoes, Berries	Naringenin chalcone, 4-Hydroxyphenylacetic acid	*Lactobacillus*, *Bifidobacterium*, *Eubacterium*	Antioxidant, anti-inflammatory, supports liver health, antineurodegenerative diseases	Cai et al. (2023), Goyal and Agrawal (2022)
Myricetin	Apple, peach, orange, pineapple, and sweet potato	Myricetin-3′-O-sulfate, dihydromyricetin	*Akkermansia*, *Ruminococcus*, Parabacteroides	Anti-ulcerative colitis (UC), antioxidant	Balázs et al. (2023), Miao et al. (2021), Yao et al. (2019)

### 4.4 Modulation of polyphenol-gut microbiota interaction

The mechanism of action of polyphenol-gut microbiota interaction is based on the mutual, bidirectional dynamic relationship between polyphenols and gut microbiota. These metabolites have been recognized in a wide sense to play a considerable role in shaping gut microbiota composition, selectively promoting the growth of beneficial bacteria like *Bifidobacteria* and *Lactobacilli*, while inhibiting the growth of pathogenic species such as *Clostridium* and Enterobacteriaceae (Krishnamurthy et al., 2023). This kind of selective modulation favours healthier balance and improved microbial diversity. In turn, the gut microbiota metabolizes polyphenols into bioactive metabolites such as phenolic acids and urolithins, which eventually exerts systemic effects including antioxidant, anti-inflammatory, immunomodulatory and cardioprotective actions. This interaction indirectly modulates host metabolic pathways through microbial metabolites by influencing gut barrier integrity, lipid metabolism, and glucose homeostasis (Sarubbo et al., 2023). The mechanisms involved in various health outcomes for gut-microbiota and polyphenol interactions is summarized in Table 4.

**TABLE 4:** Health outcome-based functions and mechanisms involved in gut-microbiota and polyphenol interactions.

Health outcomes	Biodynamic function(s)	Mechanism(s) (metabolic pathways/Signalling and modulators) involved	Effects	Study design	Species	Dose/Formulation	Main outcome	References
Cardiovascular health	Fermentation and metabolite production	Decreased production of trimethylamine (TMA) and trimethylamine N-oxide (TMAO)	1) Decreases cholesterol deposition 2) Reduces risk of atherosclerosis and cardiovascular events	Review	Human/Animal models	Not Specified (Review)	Summarizes evidence on how gut microbiota metabolites like TMAO influence cardiovascular disease risk	Cho et al. (2017), Simó and García-Cañas (2020)
		Increased production of SCFAs	1) Lowers high blood pressure 2) Reduces inflammation	Randomized, double-blind, placebo-controlled, crossover trial	Humans (healthy men and women)	100 mg/day epicatechin; 160 mg/day quercetin	Quercetin and epicatechin improved endothelial function	Mishima and Abe (2022), Verhaar et al. (2020), Wang et al. (2019)
		Bile acid metabolism (primary to secondary)	1) Modulates cholesterol metabolism 2) Reduces risk of atherosclerosis	*In vitro* study	Cardiac fibrosis cell model	Ellagic acid and punicic acid (10 µM)	Showed anti-oxidant and anti-inflammatory effects, reducing markers of cardiac fibrosis	Kriaa et al. (2019)
	Barrier integrity and gut function	Decreased gut permeability (leaky gut) and prevention of translocation of bacterial endotoxins such as lipopolysaccharides (LPS)	1) Reduces risk of atherosclerosis, endothelial dysfunction and vascular damage 2) Decreases systemic inflammation	Animal study	Rats (with chronic kidney disease)	Rutin (50 mg/kg/day)	Improved kidney and heart structure and function; reduced inflammation and fibrosis	Alfaddagh et al. (2020), Di Vincenzo et al. (2024)
	Immune modulation and inflammation	Decreased pro-inflammatory immune responses	Regulates blood pressure	Animal study	Spontaneously Hypertensive Rats (SHR)	Catechin (20 mg/kg/day)	Reduced blood pressure, associated with modulation of arachidonic acid metabolism	Richards et al. (2017)
Diabetes and obesity	Metabolite production	Increased production of SCFAs	1) Improves insulin sensitivity 2) Increases glucose metabolism	Cross-sectional study	Human (Chinese population)	Dietary myricetin intake (assessed via food frequency questionnaire)	Higher myricetin intake was inversely associated with the prevalence of type 2 diabetes	Blaut, (2015)
		Bile acid metabolism	1) Modulates lipid metabolism and glucose balance 2) Modulates branched-chain amino acids (BCAAs) and reduces insulin resistance and obesity	Review	Human/Animal models	Not Specified (Review)	Discusses naringenin’s potential to improve glucose and lipid metabolism	Cai et al. (2022)
	Barrier integrity	Decreased gut permeability (leaky gut) and prevention of entry of LPS into blood stream	1) Decreases insulin resistance and incidence of type 2 diabetes 2) Decreases systemic inflammation	Animal study	Rats (streptozotocin-induced type 2 diabetic)	Sitagliptin (10 mg/kg/day)	Ameliorated microbial dysbiosis and enhanced gut barrier integrity	Fasano (2020)
	Hormonal regulation	Increased release/secretion of glucagon-like peptide-1 (GLP-1) and peptide YY (PYY)	1) GLP-1 aids in insulin secretion, glucose metabolism and appetite control 2) Suppresses appetite and promotes weight loss	Animal study	Mice (high-fat-induced obesity)	Resveratrol (0.4% w/w in diet)	Resveratrol modified gut microbiota and reduced fat storage	Fetissov (2017), Kato et al. (2021)
Inflammation and infections	Metabolite production	Increased production of SCFs	1) Inhibits activation of nuclear factor kappa-light-chain-enhancer of activated B cells (NF-kB) and regulates inflammatory responses 2) Regulates secretion of anti-inflammatory cytokines (stimulates IL-10) and pro-inflammatory (inhibits TNF, IL-6) cytokines and reduces inflammation	*In vitro* gut model (SHIME)	Human gut microbiota	Polyphenols from black tea and red wine/grape juice	Polyphenol metabolism was source-specific, modulating microbial community and activity	Dinetz et al. (2024), Parada Venegas et al. (2019), Rekha et al. (2024)
	Gut barrier function	Decreased gut permeability (leaky gut) and prevention of entry of LPS into blood stream Increased expression and function of tight junction proteins	Decrease gut permeability and reduce inflammation	Animal study	Mice (with ulcerative colitis)	Myricetin and M10 (a derivative) (50 mg/kg)	Modified composition of gut microbiota, improving colitis symptoms	Fakharian et al. (2023)
	Interactions with pathogens	Production of antimicrobial metabolites	Decreases resistance of pathogenic microbes (*Clostridium difficile, Enterococcus faecalis* and *Escherichia coli*) and reduces inflammation and opportunistic infections through activation of immune cells such as macrophages and neutrophils	*In vitro* antimicrobial assay	Bacterial and fungal cultures (*C. acnes*)	Thermophilin 110 (bacteriocin) and Resveratrol	Showed antimicrobial activity against the opportunistic pathogen *Cutibacterium* acnes	Buonomo and Petri (2016), Pickard et al. (2017)
Brain functions	Neurotransmitter production	Increased production of producing various neurotransmitters, like serotonin (5-HT), dopamine, and gamma-aminobutyric acid (GABA)	Affects brain function and behaviour and exert calming effects in neurodegenerative diseases	Animal study	Mice	Polyphenols (in context of gut microbiota)	Microbiota metabolites modulated the Th17/Treg imbalance, promoting resilience to stress-induced anxiety- and depressive-like behaviours	Dicks, (2022)
	Metabolite production	Increased production of SCFs	1) Modulates microglial activation and reducing neuroinflammation 2) Neuroprotective properties against multiple sclerosis (MS) and neurodegenerative disease like Alzheimer’s disease (AD) and Parkinson’s disease (PD)	Review	Animal/Human models	Not Specified (Review)	Comprehensive review of naringenin’s neuroprotective effects, including anti-inflammatory and anti-apoptotic mechanisms	Koh et al. (2016)
		Decreased gut microbiota derived LPS into blood stream	Enhances metabolism and clearance of amyloid-beta peptides, promoting neurodegenerative processes	Animal study	Gnotobiotic mice	Polyphenols (Grape Seed Polyphenolic Extract - GSPE)	Gut microbiota is required for the bioavailability and metabolism of polyphenols, influencing their bioactivity in the brain	Choi and Mook-Jung (2022)
	Immune system modulation and autoimmune mechanism	Decreased levels of pro-inflammatory cytokines and pro-inflammatory T cells	Serves protective functions against AD and MD and also against MS	Review	Animal/Human models	Not specified (Review)	Discussed the prospective therapeutic role of naringenin for Alzheimer’s and Parkinson’s disease	Megur et al. (2020), Srivastava et al. (2022), Wu and Wu (2012)
	Vagus nerve signaling	Increased stimulation of vagus nerve	Modulates stress response and emotional regulation	Review	Animal/Human models	Not Specified (Review)	Summarizes the role of the *vagus* nerve in the gut-brain axis	Carabotti and Severi (2015)

There are some mechanistic relationships between polyphenols and gut microbiota that, nevertheless not completely understood, suggest a strict linkage underlying their metabolic functions. They exhibit a bidirectional interaction, throudh which gut microbiota metabolizes polyphenols into bioactive compounds (e.g., urolithins, equol), which then further modulate the microbial community (Corrêa et al., 2019), and a “duplibiotic” action, as polyphenols exhibit both prebiotic-like effects (stimulating beneficial bacteria) and antimicrobial effects (inhibiting pathogens) (Rodríguez-Daza et al., 2021).

Some interesting behaviors, but which are still in the field of emerging research, are strain-specific responses and host-dependent effects. Concerting the first one, selective bacterial promotion may occur, when specific polyphenol sources consistently promote distinct bacteria (e.g., pomegranate increases *Lactobacillus*; cocoa boosts *Faecalibacterium prausnitzii*) (Liu X et al., 2025). It may be mentioned that there are either metabolizing or opportunistic bacteria that act with these compounds. Some bacteria (e.g., *Gordonibacter*) directly metabolize polyphenols, while others (e.g., *Akkermansia muciniphila*) are “opportunistic,” thriving in the altered environment (Rodríguez-Daza et al., 2021). Taking into account the second emerging topic, it is worth to mention the interindividual variability and metabotypes: individuals are classified into metabotypes (e.g., equol producers vs. non-producers; urolithin metabotypes A, B, 0) based on their gut microbiota’s metabolic capacity (Liu X et al., 2025; Mahdi et al., 2025). There is also an influence of host genetics, when host gene polymorphisms (e.g., in enzymes for polyphenol conjugation) can affect an individual’s metabolic response to polyphenol intake (Scott et al., 2022b).

## 5 Limitations and future directions

During recent decades, polyphenols have gained considerable attention as naturally occurring plant metabolites that can modulate gut microbiota and contribute to health. However, how far and consistently these health benefits are realized is burdened by several limitations that lower their effectiveness.

Upon ingestion, only a small portion undergoes absorption in the upper gastrointestinal tract, whereas most pass into the colon and are metabolized into bioactive metabolites by gut microbes. This microbial metabolism is highly individual-specific (genetic, age, life style, diet, environmental exposure, etc.) since it depends on the composition and functional capacity of an individual’s gut microbiota. Such factors influence significantly polyphenol’s bioavailability and metabolism by gut microbiota and hence resulting health outcomes.

Besides, polyphenols are consumed as part of complex foods, and their interactions with the food matrix can affect their bioavailability and functions. A common problem in studies involving natural products and their biological effects is the influence of matrix effects (such as fiber, fats, carbohydrates, and proteins) on this activity. Polyphenols can very often be found in the form of conjugates with carbohydrates, which is a way for the plant to fix these natural metabolites. An example of this is delphinidin, which occurs in vegetables such as black beans in the form of an O-glycoside (Kumari et al., 2025). The association with these carbohydrates has a positive effect on the absorption of polyphenols by the organism that consumes them, which otherwise do not exhibit good interaction with membranes. Other matrix components can positively or negatively affect the bioavailability of the compounds, such as fats, which facilitate their incorporation into mixed micelles, a necessary step for intestinal absorption (a positive effect), but can hinder their digestion and form eventual aggregates with mineral deposits, such as calcium ones (a negative effect). Fiber, on the other hand, can constitute a physical barrier between intestinal enzymes and nutrients present in food, which is undesirable, but it contributes as a substrate for gut microbes, producing short-chain fatty acids (SCFAs) that have systemic health benefits, including effects on glucose and lipid metabolism (Kamiloglu et al., 2021). Therefore, our approach to the study of polyphenols should include the role of the biological matrix, which is usually positive, exerting a synergism necessary for better absorption of these compounds. Consideration should be given to the form of administration, whether in the form of plant extracts, where these effects are more pronounced, or in the form of isolated polyphenol supplements, in which appropriate excipients are chosen to maximize absorption and biological effect.

Fermentation (enzymatic metabolic degradation) by gut bacteria may, for example, depend on dietary fiber. Again, fats can also alter the solubility and absorption of polyphenols. It is for this reason that the interactions involving polyphenols make it hard to predict either its way through the digestive system or how well it will affect gut microbiota. Furthermore, there is a yet incomplete understanding of long-term impact that intake of polyphenols can cause gut microbiota. Most of the microbes could counteract the antimicrobial aspects of certain polyphenolic metabolites responsible for disturbing its balance. Such adaptation would affect the health-related effects of very long-term consumption of polyphenols or it may lead to dysbiosis. The incompleteness of research makes our understanding of polyphenols’ influence on gut microbiota even more difficult.

The dose-response relationship is another factor that may affect the health outcome of polyphenols. Usually, polyphenols have dose-dependent effects, though the optimal dosage is difficult to ascertain. While high doses can sometimes exert toxic effects, such as damaging the gut barrier or inducing oxidative stress, low doses may not elicit a sufficient response to produce measurable benefits. Despite the evident health benefits of polyphenols, consideration should be given to determining the most appropriate dosages in order to avoid adverse problems. Regarding the conflict between antioxidant and pro-oxidant activity, appropriate dosages have the benefit of neutralizing harmful free radicals (ROS), protecting cellular components from oxidative damage (Rudrapal et al., 2022a). In high concentrations, however, polyphenols can generate oxidative stress and induce apoptosis; this pro-oxidant effect is investigated for selectively targeting damaged cells, like UVA-damaged keratinocytes, but its systemic impact is complex (Lecci et al., 2021). Concerning gut microbiota modulation, adequate dosages provide prebiotic behaviour, selectively promoting beneficial bacteria and increasing microbial diversity, leading to production of beneficial metabolites (Plamada and Vodnar, 2021). High dosages may disrupt microbial balance; excessive amounts can exert antimicrobial pressure, potentially reducing microbial richness. Interindividual variability in gut microbiota means the same dose can have different effects (Rossi et al., 2025; Scott et al., 2022b).

Endocrine and thyroid function of polyphenols are also susceptible to dosage issues. Phytoestrogens like genistein and daidzein may offer benefits for cardiovascular health and menopausal symptoms at low concentrations. Notwithstanding, polyphenols can act as endocrine disruptors; some polyphenols are phytoestrogens that may antagonize estrogen receptors at high doses, potentially disrupting reproductive health. Flavonoids like quercetin can also inhibit thyroid peroxidase, disrupting thyroid hormone synthesis (Quesada-Vázquez et al., 2024). After ingestion, polyphenols are metabolized by gut microbiota and liver into various bioactive metabolites. However, low native bioavailability means high oral doses are often needed to achieve efficacy, increasing the risk of gut-level adverse effects. The same metabolic processes can also produce inactive or potentially toxic metabolites.

Many polyphenols exhibit hormetic effects, meaning low doses induce beneficial, adaptive stress responses (e.g., mild oxidative stress that strengthens cellular defense systems), while high doses can cause damaging toxicity (Leri et al., 2020). Another relevant point is that the relationship between polyphenols and the gut microbiome is a two-way track: gut microbiota determines the bioavailability and bioactivity of the polyphenols consumed, transforming them into more absorbable and often more potent metabolites. In return, the polyphenols shape the composition and function of your microbial community. This symbiotic relationship is highly individual and is a key area for future “precision nutrition”. Besides, the health benefits observed from consuming whole, polyphenol-rich foods are likely due to the synergistic action of multiple compounds rather than a single, high-dose polyphenol. Hormetic and synergistic effects are described in the literature, for examples, with luteolin (Abbasi et al., 2014) and ferulic acid (Moghadam et al., 2018). This natural combination may provide a balanced effect that is difficult to replicate with isolated supplements.

There is a lack of standardization in experimental design, polyphenol sources, dosages, and forms-for example, extracts, purified metabolites, or whole foods-used, and also studied populations-human, animal, or *in vitro* models-lead to a lack of coherent results. In this context, dosing, formulation, and delivery of polyphenols need to be optimized and developed in order to achieve their optimal bioavailability and effective bio-functionality. In spite of these hurdles, polyphenols may still be considered one of the most promising areas in gut health research.

Some contradictions and knowledge gaps about polyphenols and their role on gut microbiota that as mentioned in the literature are the long-held hypothesis suggesting that obesity is linked to an increased firmicutes/bacteroidetes ratio, and that polyphenols can reverse this, what is not a consensus (Corrêa et al., 2019). Besides, a significant knowledge gap exists in identifying the full repertoire of bacterial enzymes (PAZymes) involved in polyphenol metabolism and their regulation (Rodríguez-Daza et al., 2021). Furthermore, the ecological consequences of the “duplibiotic” effect is not fully elucidated. A weak understanding about how the correlations between polyphenol intake and microbial changes can be translated in a huge causality in human health outcomes is still challenging (Scott et al., 2022b).

Personalized nutrition approaches that consider individual microbiota profiles and dietary habits might overcome such limitations. Well-designed dose optimization studies with adequate methodological standardization might further show a precise role of polyphenols in gut microbiota modulation. Overcoming these limitations may realize the full therapeutic potential of polyphenols in maintaining gut health and disease management.

Future directions on the research must face precision nutrition and personalization, taking into account a person’s gut microbiota composition and metabotype in order to allow for tailored dietary recommendations with specific polyphenols to achieve desired health outcomes. Furthermore, closing the mechanistic knowledge gaps requires integrating multi-omics approaches (genomics, metabolomics, proteomics) with advanced computational modeling, that shall help identifying key bacterial strains, enzymes, and bioactive metabolites, moving from observational to predictive science (Liu X et al., 2025; Zeb et al., 2024).

While planning future research directions on exploring the relationship between dietary polyphenols and gut microbiota, several important tasks should be pursued in order to go deep into the understanding of all its potential for achieving human health and protective against diseases. Among them, mechanistic insight concerning polyphenol-microbiota interactions is the of utmost importance. Several bioactive metabolites arising from polyphenols processed by gut microbes have gained attention due to their apparent health-related benefits; however, the microbial species driving these conversions and pathways are not yet well-characterized. The aim of future research should focus on the interactive mapping of polyphenolic intake and gut microbiome, pointing to important classes of bioactive metabolite production and further to oversee how such metabolites can then affect host cell pathways towards better intestinal and non-intestinal health and associated immune-inflammatory and other biological responses. Crosstalk understanding at the molecular level between polyphenols-gut microbiota and a host cell may lead scientists to identify new targets for curative therapies. The second, very important sector involves elaboration on personalized nutrition approach-the personal variations in the composition of gut microbiota contribute so much to modifying bioavailability, metabolism and the efficacy of polyphenols.

The use of animals in testing bioactive compounds has been the subject of some controversy over the years, not only due to ethical aspects, but also because of the limitations of mimicking the biological response (including toxicology) of a target organism based on results from another species with different physiology. For example, there is the difficulty in simulating the activity of compounds against neurodegenerative diseases such as Alzheimer’s using interventional animal models (Zeiss, 2015). In this context, alternatives such as mechanistic animal models have emerged. The most common mechanistic models are: animal growth models (AGM), which detail energy and mass balance to predict individual growth based on supply and metabolic costs; animal migration models (AMM), which combine behavioral elements like foraging, flight, and fuel use to explain migration patterns; and metabolic models (MM), which integrate high-throughput data to understand metabolism in microbial communities and pathogens, and to identify drug targets. Despite the usefulness of the approach, some drawbacks must be considered (Hartung, 2024): animal models often fail to predict drug efficacy and safety in humans, leading to false negatives (abandoning potentially good drugs) and false positives (drugs that work in animals but fail in humans). Several factors limit the accuracy of animal models, including biological differences, artificial experimental conditions, and study design limitations. Adverse events in humans are sometimes missed by animal testing. It is necessary to supplement animal research with more reliable human-based techniques like organs-on-chips, computer models, and big data analysis.

## 6 Conclusion

In conclusion, the interplay between dietary polyphenols and gut microbiota represents a multifaceted nexus with far-reaching implications for human health and diseases. This review summarized a comprehensive understanding the topic, overseeing the mechanistic underpinnings of gut microbiota-polyphenol interactions and their implications in maintaining human health and disease management with future research scopes and directions within the purview of rigorous scientific inquiry. Understanding the complex interactions between dietary polyphenols and gut microbiota metabolism is essential for developing targeted dietary interventions and functional foods aimed at enhancing health and preventing development of various diseases. Several such interventions targeting the gut microbiota milieu through strategic dietary manipulations, probiotic formulations and prebiotic supplements hold considerable promise in promoting health and amelioration of diverse disease conditions. However, more research would further help elucidate the exact molecular mechanisms of polyphenol-gut microbiota interactions and the physiological effects of microbiota-derived polyphenol metabolites on human health. Understanding such intricate biological interactions between gut-microbiota and polyphenols can unfold the potential of polyphenol-rich diets in influencing human health and disease outcomes.
